# Role of pre-operative dexamethasone as prophylaxis for postoperative nausea and vomiting in laparoscopic surgery

**DOI:** 10.4103/0972-9941.25671

**Published:** 2006-03

**Authors:** P Gupta, J Khanna, A K Mitramustafi, V K Bhartia

**Affiliations:** Fellow National Board-Minimal Access surgery, Institute of Minimally Invasive Surgery, AMRI Hospitals, Kolkata, India; 1 Head - Department of Anaesthesia, Institute of Minimally Invasive Surgery, AMRI Hospitals, Kolkata, India; 2 Director - Institute of Minimally Invasive Surgery, Institute of Minimally Invasive Surgery, AMRI Hospitals, Kolkata, India

**Keywords:** Laparoscopic surgery, steroids, preoperative dexamethasone, postoperative nausea and vomiting

## Abstract

**Introduction:**

Laparoscopic surgery provides tremendous benefits to patients, including faster recovery, shorter hospital stay and prompt return to normal activities. Despite the minimally invasive nature of laparoscopy, high incidence of postoperative nausea and vomiting remains a major cause for morbidity. The aim of the present study was to investigate whether preoperative Dexamethasone can reduce PONV in patients undergoing laparoscopic Surgery.

**Materials and Methods:**

The study included 200 patients undergoing laparoscopic cholecystectomy. We divided the patients into two groups; one group received preoperative Dexamethasone (group 1) and the other group received Ondansetron (group 2). After surgery, patients were observed for any episode of nausea or vomiting, or whether the patient required any anti-emetic drug in the postoperative period.

**Results:**

The two groups, (Dexamethasone and Ondansetron) were comparable in outcome, in terms of post-operative nausea and vomiting, in patients undergoing laparoscopic cholecystectomy. In group I, 24% of patients had nausea, as compared to 30% in group II (*P*=0.2481). Similarly, 12% of patients in group I and 18% of patients in group II had vomiting (*P*=0.3574).

**Conclusion:**

We conclude that, preoperative intravenous low dose Dexamethasone reduces the incidence of PONV and is comparable to intravenous Ondansetron.

Despite the introduction of new anti-emetic drugs, short-acting anaesthetic agents and minimal invasive surgical techniques, the incidence of postoperative nausea and vomiting (PONV) has remained largely unchanged. Use of anti-emetic prophylaxis has become the standard approach to minimize the nausea and vomiting postoperatively.

Glucocorticosteroids are well known for their analgesic, anti-inflammatory, immune- modulatory and anti-emetic effects. Several clinical trials have been conducted to determine the effects of glucocorticoids on surgical outcome.[1]

The aim of the present study, was to investigate whether preoperative Dexamethasone can reduce PONV in patients undergoing laparoscopic Surgery. We also compared the anti -emetic effects of Dexamethasone with an established anti emetic drug, Ondansetron.

## MATERIALS AND METHODS

This is a prospective randomized study on patients undergoing laparoscopic cholecystectomy (LC) between October 2004 to January 2005. We obtained the approval from the hospital ethical committee and got the informed consent from the patients for the following study. Patients with ASA Grade III/IV, Mentally ill health, GERD patients, Pregnancy or having h/o motion sickness, were excluded from the study.

We divided the patients into 2 groups depending on their hospital inpatients number (INP). Patients with even number INP were categorized in group one and those in odd number INP were group two. Group one patients received IV Dexamethasone (5 mg) and group two received IV Ondansetron (4 mg), 90 minutes prior to induction of anaesthesia. This was administered by a house-staff, who himself administered the drugs to blind the anaesthetist and the surgeon.

The patients received single dose of injections (Amoxicillin+Clavulanic acid) at the time of induction of anaesthesia. All patients received Inj Diclofenac 50mg i/m thrice daily for day 1 and then as and when required. If patients developed nausea or vomiting, they were given injection Ondansetron 4 mg intravenously, irrespective the group.

### Surgery and anaesthesia

All the patients received similar type of standardized anaesthetic regimen - induction with Propofol, maintenance with Isoflurane, relaxation with Atracuronium and reversal with Neostigmine. Nasogastric tube was placed in every patient to empty the stomach of air and gastric contents, which was taken out as a routine, at the end of the procedure.

Reverse Trendelenberg with right side up position was made and pneumoperitoneum was created at the supraumbilical port site, with the closed needle technique. During laparoscopy, intrabdominal pressure (IAP) was maintained at 12 mm Hg and at the end of surgery, Co_2_ was removed by suction and manual compression of abdomen. LC was preformed using standard 4 ports technique with two 10 mm and two 5 mm ports. Gall bladder was extracted through the epigastric port and the 10 mm port fascial defect was closed with 3/0 vicryl.

After surgery, patients were observed for any episode of nausea or vomiting, or whether the patient required any anti-emetic drug in the postoperative period. Patients were evaluated for nausea and vomiting in the first 24 hrs. Nausea was rated on the VRS grade (Gr 0-no nausea, Gr I - mild, Gr II - moderate, Gr III - severe nausea) and the number of vomiting episodes was noted. Vomiting was graded as Gr 0-no vomiting, Gr I- less than 4 vomiting, Gr II - more than 4 vomiting. Vomiting was treated with the intravenous Ondansetron.

Patients were followed up in hospital from the day of surgery, to the day of discharge. Day of operation was defined as D0, first day was defined as D1 and so on. All the patients were discharged on D2.

The data was analyzed using Pearson's Chi square test with *P*<0.05 taken as significant.

## RESULTS

We included 200 patients in the study; 100 patients were enrolled for both the groups. The data obtained from the patients undergoing LC were analyzed. The characteristics of the patients and duration of surgery were similar between the two groups, as shown in Table 1.

**Table 1:** Characteristics of the patients

	Group I	Group II	*P value*
No. of patients	100	100	
Age	24–62 yrs (38)	28–69 yrs (40)	
Sex ratio (F:M)	61: 39	73: 27	0.0711
Duration of surgery	25–60	20–55	
	minutes (48)	minutes (46)

After surgery, patients were observed for any episode of nausea or vomiting, or whether the patient required any anti-emetic drug in the postoperative period. We graded the nausea and vomiting episodes, which is shown in Table 2. None of the patients had severe nausea.

**Table 2:** Nausea and vomiting in our patients

Grading of nausea	Group I	Group II	*P* value
Gr I- no nausea	76	70	
Gr II- mild nausea	19	26	0.2481
Gr III- moderate nausea	05	04	
Gr IV- severe nausea	Nil	Nil	
Grading of vomiting	Group 1	Group 2	
Gr 0: No Vomiting	88	82	
Gr I: <4 vomiting	11	15	0.3574
Gr II: >4 vomiting	01	03

In the first group receiving (Dexamethasone); 24 patients reported nausea and 12 had episodes of vomiting. In the other group receiving Ondansetron, 30 patients reported nausea and 18 patients had vomiting in the postoperative period. The difference in the incidence of nausea and vomiting between the two groups was comparable and statistically not significant (nausea *P*=0.2481: vomiting *P*=0.3574). Since the number of patients with grade III and IV nausea, as well as grade II vomiting was small, they were not included while performing the statistical analysis. All the patients were discharged on D2 except one patient in the Dexamethasone group, who had Gr II vomiting and he was discharged on day 4. We did not have any postoperative complication in the form of wound infection, or delayed wound healing.

## DISCUSSION

Laparoscopic surgery provides spectacular benefits to patients and LC has become the gold standard for symptomatic cholelithiasis. However, a high incidence of PONV (53–70%) has been reported.[2,3] Several agents like Metoclopromide, Prochlorperazine, Droperidol, Ondansetron etc have been used to prevent nausea / vomiting.

Dexamethasone was reported as an effective anti-emetic in patients receiving cancer chemotherapy in 1981.[4] The incidence of postoperative nausea and vomiting has been significantly decreased by preoperative single dose steroid administration in several studies.[5,6] Glucocorticoids have been recognized as an important modifier of the postoperative physiology, inflammatory, humoral and immunologic response, by regulation of trauma induced humoral mediators.

As an immune modulation strategy, Dexamethasone appears to shift the balance of inflammation, in favor of anti-inflammatory mediators. The incidence and severity of PONV have been significantly decreased as shown in several studies.[7] This prophylaxis also seemed to reduce postoperative pain and early convalescence. BisGaard *et al* concluded that, preoperative Dexamethasone reduced pain, fatigue, nausea, vomiting and duration of convalescence in patients undergoing LC, as compared to placebo and they recommend the routine use of Dexamethasone.[8]

The exact mechanism by which glucocorticoids decrease the incidence of nausea / vomiting is not fully understood, but probably can be explained by centrally mediated anti-emetic action via inhibition of prostaglandin synthesis, or inhibition of release of endogenous opoids.[9]

Recently, metanalysis of 17 randomized controlled trials have shown that Dexamethasone combined with 5HT3 receptor antagonist significantly reduces the postoperative nausea or vomiting as compared to placebo.[8,10,11] The role of the concomitant 5HT3 receptor has to be clarified.

Many studies have used preoperative Dexamethasone administration just before induction of anaesthesia.[12] Glucocorticoids act on the intracellular receptor and the effects are mediated through altered prostaglandin synthesis, via gene transcription. Onset of biologic action of glucocorticoids is 1–2 hours.[13,14]

Since action of early mediators of metabolic response to surgery occurs immediately after surgical incision, it seems appropriate to administer Dexamethasone 1–2 hours preoperatively, to achieve full postoperative benefits of the treatment. In the present study, we administered the IV Dexamethasone, 90 min before skin incision was made. We all know that metabolic response to surgery starts immediately as soon as the skin incision is made.

The timing of steroid administration seems to be the key (1–2 hr preoperatively), if excess inflammatory and related postoperative morbidity is to be attenuated.

The major concern regarding the use of Dexamethasone is infection, delayed wound healing and other side effects. But various studies in the literature have shown that single-dose Dexamethasone does not increase complications.[15–17] A recent metanalysis concluded that, perioperative administration of high dose of Methylprednisolone (30–35 mg/kg), a dose approximately 50 times that of the dose used in the study, was not associated with significant side effects.[18] We did not have any postoperative complication which could be attributed to Dexamethasone prophylaxis.

In our study, we found that incidence of PONV was 24 and 30% in patients receiving IV Dexamethasone and Ondansetron, respectively.

## CONCLUSION

We have an opportunity to enhance further, the benefits of minimally invasive surgery.

Long-term corticosteroids may have significant morbidity. A single dose of Dexamethasone is safe and is comparable to Ondansetron. We conclude that, preoperative intravenous low dose Dexamethasone reduces the incidence of PONV and is comparable to intravenous Ondansetron.
